# High dietary fat intake lowers serum equol concentration and promotes prostate carcinogenesis in a transgenic mouse prostate model

**DOI:** 10.1186/s12986-019-0351-x

**Published:** 2019-04-11

**Authors:** Yufei Liu, Xiaobo Wu, Haowen Jiang

**Affiliations:** Department of Urology, Huashan Hospital, Fudan University, No. 12 Middle Wulumuqi Road, Shanghai, 200040 China

**Keywords:** Prostate cancer, High fat diet, Gut microbiota, Daidzein, Equol

## Abstract

**Background:**

Consumption of diet high in soy products is suggested to contribute to lower prostate cancer incidence in Asian men. But little has been known about the influences of dietary patterns on gut microbiota and microbiota-mediated isoflavone metabolism. Here, we determined the influences of western pattern diet on prostate carcinogenesis, gut microbiota and microbiota-mediated equol metabolism using a transgenic adenocarcinoma of mouse prostate (TRAMP) model.

**Methods:**

We mimicked the western pattern diet using a high fat diet (HFD). TRAMP mice were fed with either control diet (CD) or HFD. At the age of 24 weeks, mice were orally administered daidzein over a 4-day period, and then sacrificed. The serum daidzein and equol were analyzed by ultra high performance liquid chromatography. Fecal microbiome was analyzed with fecal 16S rRNA pyrosequencing, and prostate was dissected and performed with histopathology.

**Results:**

HFD could promote prostate carcinogenesis in TRAMP mice (*p* = 0.045). The daidzein showed no significant differences between CD and HFD groups, while equol was significantly decreased in HFD group (*p* = 0.019). Fecal microbiotas differed between the two groups, 21 microbial phylotypes were increased and 11 phylotypes were decreased in abundance in HFD group, including decreased abundance of equol-producing bacterium *Adlercreutzia* (0.08% vs. 0.27%)*.*

**Conclusions:**

HFD may promote prostate carcinogenesis through adversely affecting equol-producing bacterium. Further functional validations are required to ascertain the mechanism of those HFD-responsive bacteria in carcinogenesis.

## Introduction

Prostate cancer (PCa) is the most frequently diagnosed cancer in men worldwide, with an estimated 1.1 million cases and 307,500 deaths globally in 2012 [1]. There is a noticeable discrepancy in PCa incidence rates between Asian and Western countries. In addition to ethnic and genetic factors, consumption of diet high in soy products is suggested to contribute to lower PCa incidence in Asian men [2, 3].

Isoflavones, including daidzein and genistein, are commonly contained in soy products. Numerous studies have described isoflavones’ protective role against PCa due to their estrogenic properties [4, 5]. For example, daidzein could induce cell-cycle arrest of prostate cancer cell in G0/G1 phase via impacting the gene expression of cyclins and cyclin-dependent kinases (CDKs) [6]. Equol is secondary metabolite of daidzein produced by the intestinal microbiotas with stronger anti-carcinogenic activity than daidzein. According to a 14,203 Japanese men based study conducted by Norie Kurahashi et al., plasma equol was significantly associated with a decreased risk of PCa, especially localized cancers [7]. Equol normally exists as a diastereoisomer but intestinal bacteria synthesize exclusively the S-(−)equol enantiomer, not R-(+)equol. The S-(−)equol has selective affinity for the estrogen receptor-β [8]. What’s more, not all men and women are equol-producer, 30–50% of adults lack those equol-producing bacteria to metabolite daidzein to S-equol.

Bidirectional interactions exist between diet and gut microbiota, diet influences the profile of microbiota, and microbiota inversely affects the metabolism of dietary elements. Available studies are limited to the observation of the discrepancy of PCa incidence between Asian and Western countries, possibly because of soy consumption, but few have investigated the influences of dietary patterns on gut microbiota and microbiota-mediated equol metabolism. In the present study, we mimicked the western pattern diet using high fat diet (HFD), and observed the effects of HFD on S-equol metabolism and prostate carcinogenesis using a transgenic adenocarcinoma of mouse prostate (TRAMP) model. We also examined the variation of gut microbiota under HFD consumption that may be associated with equol production.

## Methods and materials

### Materials

Daidzein (D-2946) was purchased from LC laboratories (Woburn, USA); S-(−)equol (SML2147) was purchased from Sigma-Aldrich (Germany); Equol-d4 (E593002) was purchased from Toronto Research Chemicals (TRC, Canada).

### Animals

Animals were handled with IACUC approval by the Fudan University (Grant No. 2017 1674 A641). Transgenic TRAMP females (purchased from Jackson Laboratory, Bar Harbor, USA) were bred with nontransgenic C57BL/6 males (Jackson Laboratory). At age of 2 weeks, the transgenic males were selected by genotyping as previously reported. TRAMP mice were randomly categorized into two groups after weaning (postnatal day 20) and singly housed and fed either control diet (CD, *n* = 6) (16% of calories from fat, SLAC Inc., http://www.slaccas.com; Shanghai, China) or HFD (n = 6) (40% of calories from fat, SLAC Inc.,) (Table 1). At age of 24 weeks, daidzein was administered orally to the HFD and CD groups for 4 days. Daidzein was dissolved in DMSO. Each mouse was given 3 mg of daidzein dissolved in 0.2 ml of DMSO for the first 3 days, and 6 mg of daidzein dissolved in 0.2 ml of DMSO on the fourth day [9].

**Table 1:** Composition of HFD and control diet

Composition	HFD		Control diet	
	gm%	gm%	gm%	Kcal%
Protein	17	15	15	15
Carbohydrate	52	45	69	69
Fat	20	40	7	16
Total		100		100
Kcal/gm	4.6		4.0	

### Samples

Fecal samples were collected one day before the daidzein administration, and immediately stored at − 80 °C until gut microbiota analysis. Sixteen hours after daidzein administration on the fourth day, the mice were sacrificed. The blood was harvested from the portal vein, and the serum was collected and stored at − 80 °C until UHPLC-MS/MS analysis. The prostate was removed and fixed for histopathological analysis based on a modified grading system for prostatic lesions in TRAMP mice.

### Serum UHPLC-PRM-MS/MS analysis

A 60 μL aliquot of each individual sample was added to 180 μL methanol (containing 50 ng/mL internal standard) in an Eppendorf tube. The samples were vortex mixed for 30 s, incubated at − 20 °C for one hour and then centrifuged at 12000 rpm 4 °C for 15 min. A 200 μL aliquot of supernatant was transferred to a new tube and dried under gentle nitrogen flow. The residual was reconstituted with 50 μL of 50% methanol, centrifuged at 12000 rpm 4 °C for 15 min. A 40 μL aliquot of supernatant was transferred to an auto-sampler vial for UHPLC-MS/MS analysis.

Stock solutions were prepared by dissolving daidzein or S-equol standard substance to a concentration of 10 μg/mL. Aliquots of each stock solution were mixed to form the standard solution at 1 μg/mL. The calibration standard solutions were then prepared by stepwise dilution of mixed standard solution. Equol-d4 was used as the internal standard (IS) at a concentration of 100 ng/mL.

The UHPLC separation was carried out using an Agilent 1290 Infinity series UHPLC System (Agilent) coupled with an ACQUITY UPLC BEH C18 column (100 × 2.1 mm, 1.7 μm, Waters, Ireland). The mobile phase consisted of A = water containing 10 mmol/L ammonium acetate/ammonia, and B = methanol. The column temperature was set at 40 °C, the auto-sampler temperature was set at 4 °C, and the injection volume was 2 μL. Analysis was carried out using a Q Exactive Focus mass spectrometer (Thermo Fisher Scientific) under parallel reaction monitoring (PRM) model. The ion source parameters were: spray voltage = + 3500/− 3100 V, sheath gas (N2) flow rate = 40, aux gas (N2) flow rate = 15, sweep gas (N2) flow rate = 0, aux gas (N2) temperature = 350 °C, capillary temperature = 320 °C.

### Fecal 16S rRNA gene amplicon pyrosequencing and sequence analysis

Fecal DNA was extracted using the DNeasy PowerSoil Kit (QIAGEN, Inc., Netherlands). Regions V3–V4 of the 16S rRNA gene were amplified using the forward primer 5′-ACTCCTACGGGAGGCAGCA-3′, and the reverse primer 5′-GGACTACHVGGGTWTCTAAT-3′. The PCR program was set: 98 °C 10 min, 25 cycles of 98 °C 15 s, 55 °C 30 s, 72 °C 30 s, and 72 °C 5 min. After agarose gel electrophoresis, the PCR amplicons were purified using the Agencourt AMPure Kit (Beckman Coulter, Milan, Italy) and quantified using the PicoGreen dsDNA Assay Kit (Invitrogen, Carlsbad, CA, USA). An equimolar amplicon pool was obtained, and paired-end 2 × 300 bp sequencing was performed using the Illlumina MiSeq platform with MiSeq Reagent Kit v3 (Shanghai Personal Biotechnology Co., Ltd., Shanghai, China).

The sequencing data were analyzed using QIIME V1.8.0 package [10]. Sequences were excluded from analysis if they (a) were < 150 nucleotides in length, (b) had average Phred scores of < 20, (c) contained ambiguous bases or mononucleotide repeats of > 8 bp. Paired-end reads were aligned using FLASH [11], and operational taxonomic units (OTUs) delineation was conducted with UCLUST at a 97% cutoff [12]. A representative sequence of each OTU was selected and subjected to BLAST to assign taxonomic classification using the Greengenes 16S rRNA gene database. Alpha and beta diversity were performed using QIIME. LEfSe analysis was performed on website http://huttenhower.sph.harvard.edu/galaxy to identify differentially abundant phylotypes with both statistical significance and biological relevance [13].

### Statistical analysis

The Student’s t-test, nonparametric test and Spearman’s rank correlation coefficient were used for statistical analysis with SPSS 20.0 software. *P* < 0.05 was regarded as statistical significant.

## Results

### Overall situation of TRAMP mice

No mice died until sacrifice at the 24th week. The body weights of HFD and CD mice were 26.86 ± 0.87 and 23.48 ± 2.99 g, respectively (*P* = 0.024). The prostate weights of HFD and CD mice were 0.545 ± 1.139 and 0.076 ± 0.017 g, respectively (*P* = 0.336).

### Effects of HFD on prostate carcinogenesis

**Fig. 1:**
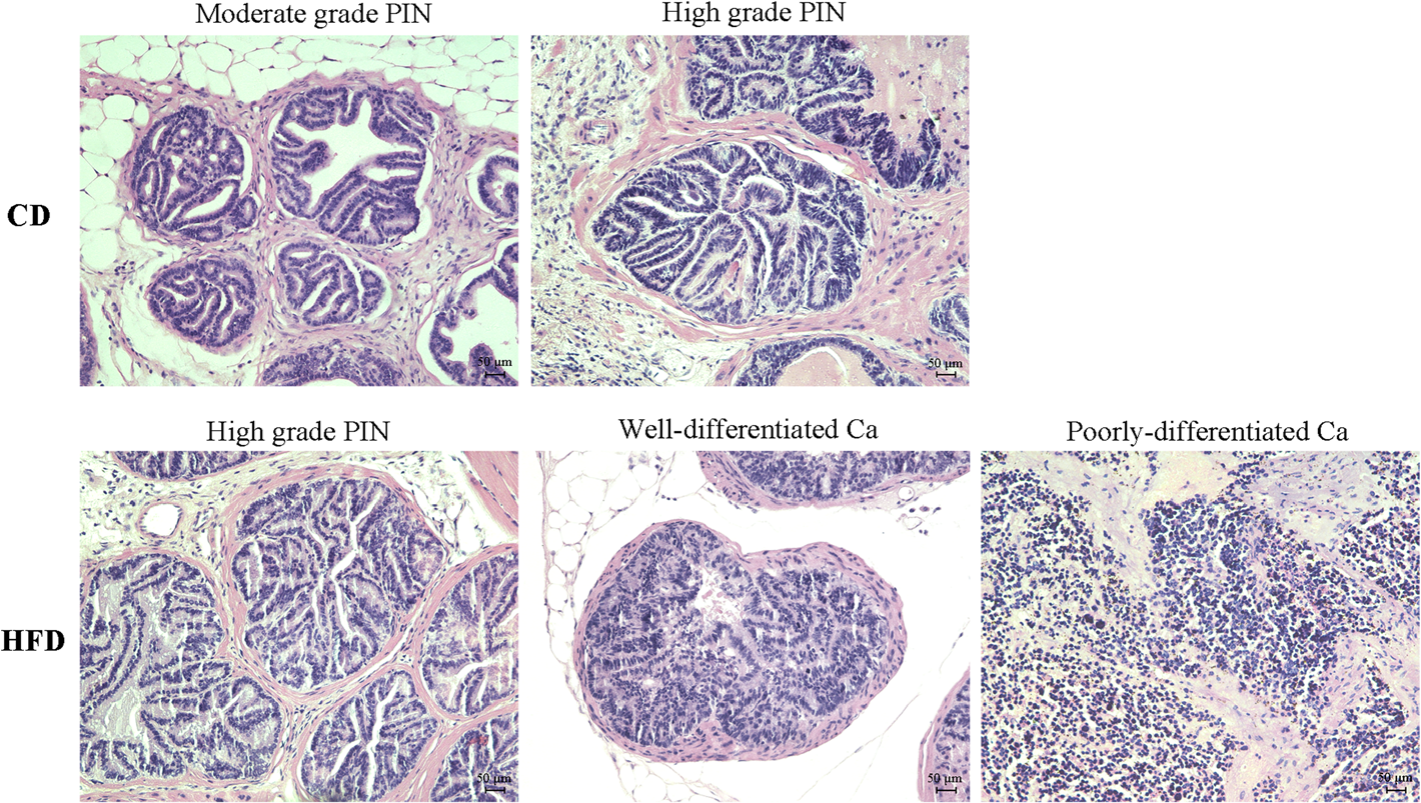
Images of histopathological diagnosis of HFD and CD mice prostate. The bar represents 50 μm

The CD group exhibited one case of moderate- and five cases of high-grade prostate intraepithelial neoplasia (PIN), while the HFD group exhibited three cases of high-grade PIN and three cases of prostate cancer (*p* = 0.045) (Fig. 1).

### Serum isoflavones

**Fig. 2:**
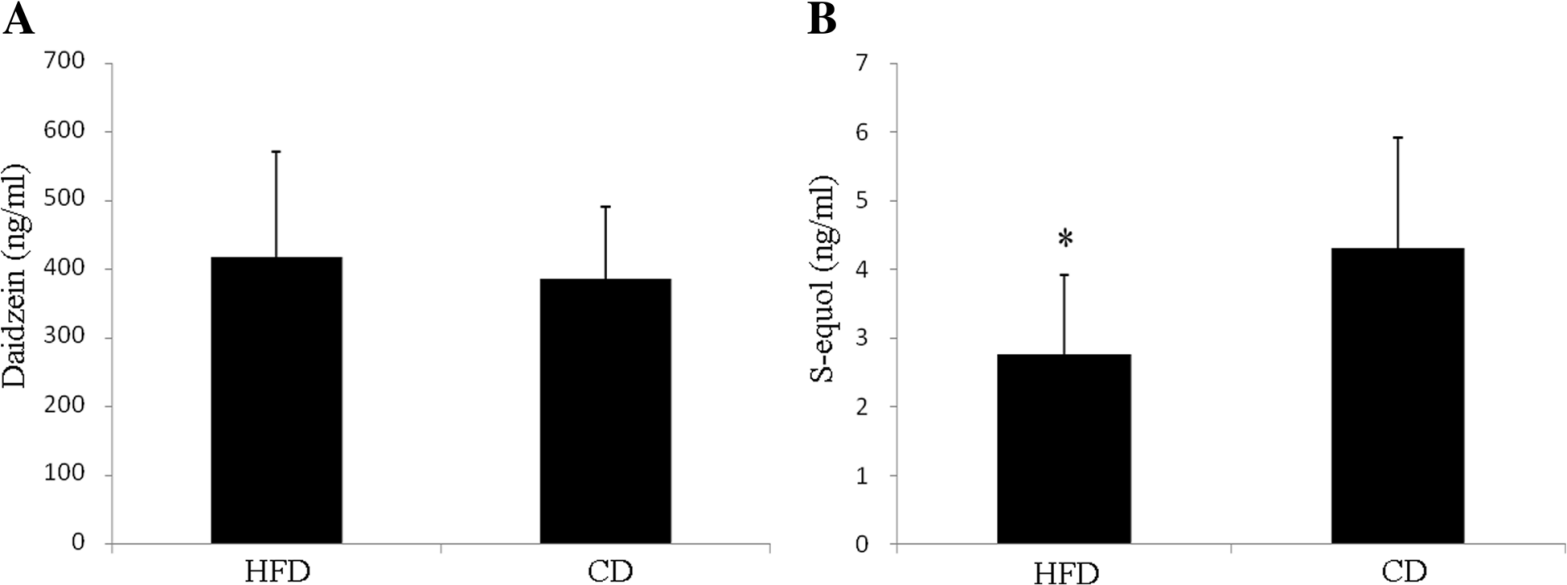
Serum concentrations of daidzein **a** and S-equol **b** in HFD and CD mice. Data were expressed as means ±SD. * indicates *p* < 0.05

The serum concentrations of daidzein and S-equol were measured. There is no significant differences in daidzein concentration between HFD and CD groups (418.21 ± 153.26 ng/ml vs. 387.16 ± 103.47 ng/ml, *p* = 0.513) (Fig. 2a). However, the S-equol concentration was significantly decreased in HFD group (2.76 ± 1.17 ng/ml vs. 4.32 ± 1.60 ng/ml, *p* = 0.019) (Fig. 2b).

### Differences in gut microbiome between HFD and CD groups

**Fig. 3:**
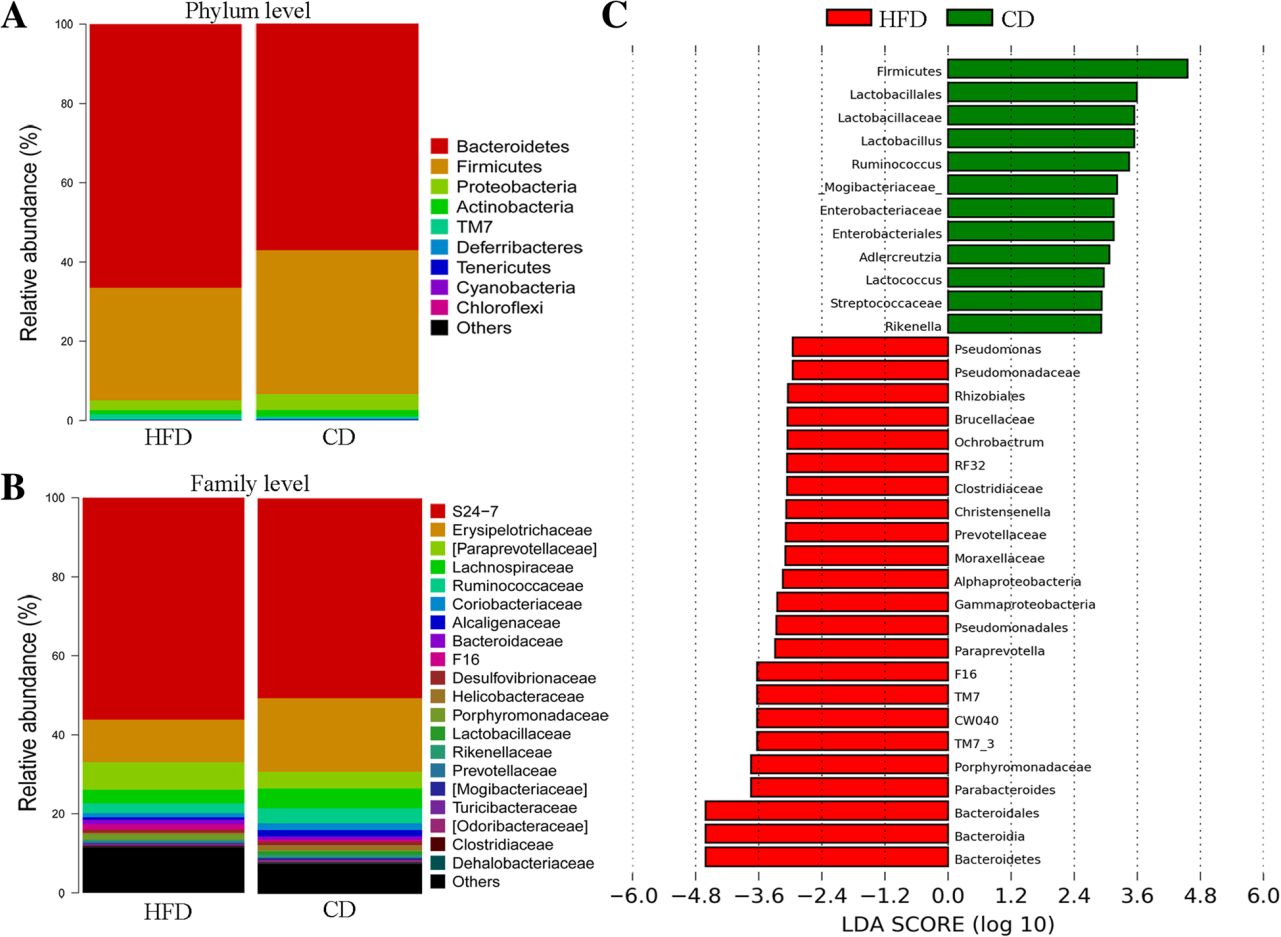
Differentiated gut microbiome between HFD and CD mice. The average relative abundance of bacterial lineages of gut microbiota within HFD and CD group at the phylum **a** and family **b** level, and the key microbial phylotypes between the two groups **c**

We profiled the gut microbiome of two groups of mice to determine whether HFD may influence the microbial populations. Alpha-diversity can indicate the richness of microbial communities. The average Shannon diversity index [14] for HFD and CD groups were 8.85 ± 0.23 and 8.98 ± 0.47 (*p* = 0.55), respectively, no statistical significances were observed between the two groups. We further determined the average relative abundance of bacteria within each group (Fig. 3a, b). At the phylum level, the HFD group had increased abundance of *Bacteroidetes* (66.53% vs. 57.10%), *TM-7* (1.33% vs. 0.45%), *Tenericutes* (0.13% vs. 0.10%); and decreased abundance of *Firmicutes* (28.37% vs. 36.29%), *Proteobacteria* (2.47% vs. 3.97%), *Actinobacteria* (1.06% vs. 1.68%), *Deferribacteres* (0.04% vs.0.30%), and *Cyanobacteria* (0.07% vs. 0.09%). The key differentiated microbial phylotypes in response to HFD intervention were identified with LefSe analysis, among which 21 phylotypes were increased in abundance in HFD group, and 11 were decreased in HFD group, including genus *Adlercreutzia*, *Ruminococcus*, *Lactobacillus*, *Rikenella*, *Lactococcus*, etc. (Fig. 3c). The detailed information was illustrated in Table 2. We also performed correlation analysis between differentiated microbial genus and the equol concentration. Significant correlations were found between equol and *Adlercreutzia* (r = 0.699, *p* = 0.011), *Ruminococcus* (r = 0.74, *p* = 0.006), and *Rikenella* (r = 0.582, *p* = 0.047).

**Table 2:** LefSe analysis of phylotypes with biologically significant differential abundance

Differential phylotypes by LefSe	Relative abundance (%)		Ratio HFD to CD
	HFD	CD	
Class			
Bacteroidia	66.527	57.100	1.17
TM7–3	1.331	0.452	2.94
Gammaproteobacteria	0.318	0.012	26.5
Alphaproteobacteria	0.316	0.085	3.72
Order			
Bacteroidales	66.527	57.100	1.17
CW040	1.331	0.452	2.94
Pseudomonadales	0.318	0	–
RF32	0.280	0.083	3.37
Lactobacillales	0.151	0.954	0.16
Rhizobiales	0.022	1.97E-05	1116.75
Enterobacteriales	0	0.012	–
Family			
Porphyromonadaceae	1.357	0.279	4.86
F16	1.331	0.452	2.94
Prevotellaceae	0.450	0.215	2.09
Clostridiaceae	0.306	0.130	2.35
Moraxellaceae	0.180	0	–
Lactobacillaceae	0.149	0.858	0.17
Pseudomonadaceae	0.138	0	–
Mogibacteriaceae	0.131	0.432	0.30
Brucellaceae	0.022	1.97E-05	1116.75
Streptococcaceae	1.97E-05	0.029	0.001
Enterobacteriaceae	0	0.012	–
Genus			
Parabacteroides	1.357	0.279	4.86
Paraprevotella	0.416	0.034	12.24
Ruminococcus	0.310	0.878	0.35
Lactobacillus	0.149	0.858	0.17
Pseudomonas	0.138	0	–
Adlercreutzia	0.080	0.278	0.29
Christensenella	0.053	3.94E-05	1345.18
Ochrobactrum	0.022	1.97E-05	1116.75
Rikenella	0	0.085	–
Lactococcus	0	0.029	–

## Conclusion

Both daidzein and its secondary metabolite equol have estrogenic properties that may contribute to protection against hormone-dependent diseases including PCa. Equol is more estrogenic than daidzein, Lu et al. reported that S-equol could inhibit the growth of LnCaP, DU145 and PC3 human prostate cancer cell lines via upregulating the Forkhead box O3 (FOXO3a) expression, a transcription factor with tumor suppressor functions in PCa. Also, S-equol inhibited the growth of PC3 xenograft tumors in BALB/c nude mice [15]. In another study led by Itsumi et al., equol was proved to be able to inhibit prostate tumor growth through degradating androgen receptor by S-phase kinase-associated protein 2 [16]. Equol exists as two distinct isomers, R-(+)equol and S-(−)equol, and the latter is the natural diastereoisomer produced by bacteria in the intestine of humans and rats. Noticeably, daidzein can also be metabolized into O-desmethylangolensin (O-DMA). Though O-DMA producer phenotype is more common than equol among the population, it is much less bioactive [17]. By far, several equol-producing bacteria have been isolated from human or animal intestines, including *slackia sp. strain NATTS, TM-40, Asaccharobacter celatus, Adlercreutzia equolifaciens* [18–20]. In our study, dietary fat intake significantly altered the mice’s gut microbiota composition, lowered the serum S-equol concentration, and promoted the prostate carcinogenesis.

Previous human and animal studies have reported the facilitative role of HFD in prostate carcinogenesis. HFD can induce prostate tumor growth through a large suite of chemical signaling pathways, such as the activation of IL6/pSTAT3 or MCP-1/CCR2 signalings, or through the inhibition of tumor suppressor gene PTEN [21–23]. In this study, HFD impaired the ability of gut microbiota to metabolize daidzein to S-equol. The relative abundance of genus *Adlercreutzia* decreased below one-third in HFD mice. Adlercreutzia *equolifaciens* has been proved to be an equol-producing bacterium that was isolated from human feces by Maruo, et al. [24]. A positive correlation between genus *Adlercreutzia* and S-equol concentration was detected in this study, indicating that the decreased *Adlercreutzia* may contribute to the lower level of equol concentration.

Several differentiated microbial floras identified in HFD mice have been proved to be associated with PCa. For example, genus *Lactobacillus* was decreased in HFD mice. Some strains in *Lactobacillus* are capable of activating NK activity of peripheral blood mononuclear cells (PBMCs) against prostate cancer cells [25]. In an PC-3 xenograft rodent model, genus *Lactobacillus* was found to be elevated in mice with suppressed tumor growth [26]. The genus *Ochrobactrum* was intensely increased in HFD mice. It was also found to be overexpressed in expressed prostatic secretions of PCa patients [27]. We also observed an increased abundance of phylum *Bacteroidetes* and decreased *Firmicutes* in HFD mice. Whether the ratio of *Bacteroidetes/Firmicutes* is associated with obesity remains controversial, for example, Turnbaugh et al. reported decreased while Schwiertz et al. reported increased *Bacteroidetes/Firmicutes* ratio in obese humans [28, 29]. Golombos et al. compared the gut microbiome between PCa patients and patients with benign prostatic hypertrophy, and found that *Bacteriodes massiliensis*, which belongs to *Bacteroidetes* was significantly increased in cancer patients [30].

No significant differences were observed in the daidzein concentration, indicating that HFD did not significantly influence the daidzein absorption. Previous studies proposed that some strains in *clostridia* may participate in isoflavone degradation and metabolism [31]. Correspondingly, no significant differences were observed in the relative abundance of *Clostridia* between HFD and CD groups (17.04% vs. 16.64%).

In summary, the present study suggested that dietary high fat intake may promote prostate carcinogenesis via adversely affecting the gut microbiota and microbiota-mediated equol metabolism. Further functional validations are needed to ascertain the exact roles of those differentiated microbial floras, and their potentials to be used as new targets for PCa prevention.

## Abbreviations

BLAST: Basic local alignment search tool
FLASH: Fast length adjustment of short reads
HFD: High fat diet
LEfSe: Linear discriminant analysis effect size
PCa: Prostate cancer
PIN: Prostate intraepithelial neoplasia
PSA: Prostate specific antigen
TRAMP: Transgenic adenocarcinoma of mouse prostate
UHPLC-MS/MS: Ultra-high performance liquid chromatography-tandem mass-spectrometry
